# Ingestion of rubber tips of artificial turf fields by goldfish

**DOI:** 10.1038/s41598-023-28672-3

**Published:** 2023-01-24

**Authors:** Rihito Chiba, Ryosuke Fujinuma, Tomoyasu Yoshitomi, Yasuo Shimizu, Makito Kobayashi

**Affiliations:** 1 Department of Natural Sciences, International Christian University, 3-10-2 Osawa, Mitaka, Tokyo 181-8585 Japan; 2 Field Studies Institute for Environmental Education, Tokyo Gakugei University, 4-1-1 Nukuikita-Machi, Koganei, Tokyo 184-8501 Japan; 3 Department of Physical Education, International Christian University, 3-10-2 Osawa, Mitaka, Tokyo 181-8585 Japan

**Keywords:** Ecology, Ecology, Environmental sciences, Environmental social sciences, Ocean sciences

## Abstract

Marine microplastics are one of the global environmental issues. The present study examined whether rubber tips of artificial sports fields could be marine microplastics. We observed the migration of rubber tips from the artificial turf field to the surrounding ditch connected to sewer pipes and then examined the ingestion of rubber tips using the goldfish *Carassius auratus*. The rubber tips found in sediments in the ditch suggest that the rubber tips could be sent to the river and released into the ocean. The goldfish ingested rubber tips with or without fish feed, and rubber tips were found in the intestine. However, the fish discharged the rubber tips within 48 h after ingestion. These results indicate that ingestion of the rubber tips was not accidental but an active behavior. Therefore, artificial turf sports fields could be a source of marine microplastics and may cause hazardous effects on wild fishes through ingestion.

## Introduction

Marine microplastics (MPs) are one of the global environmental issues requiring urgent preventive measures^1–5^. MP, a plastic particle smaller than 5 mm, has been released massively into the aquatic environment from diverse sources, such as litter, cosmetic products, textile waste, packaging, construction waste, agricultural plastic film, tires, road paint, and artificial turfs^1–5^. Furthermore, MPs were found in the digestive tracts of marine and freshwater wild fishes^6–10^. Hence, MPs are considered hazardous impacts on aquatic organisms and ecosystems^11–13^.

The presence of MP in commercial fish, in particular, raised concerns regarding human health^14–18^. Laboratory experiments showed the ingestion of MPs in diverse fishes, such as yellow croaker *Larimichthys crocea*^19^, white seabream *Diplodus sargus*^20^, mummichog *Fundulus heteroclitus*^21^, fathead minnow *Pimephales promelas*^21^, intertidal fish *Girella laevifrons*^22^, largemouth bass *Micropterus salmoides*^23^, yellow catfish *Pelteobagrus fulvidraco*^23^, bighead carp *Aristichthys nobilis*^23^, goldfish *Carassius auratus*^23,24^, and African catfish *Clarias gariepinus*^25^. Furthermore, ingested MP induces lesions and inflammation of the intestine and changes in intestinal microbiota^26,27^. Therefore, marine MP could cause a decrease in the population size of marine fishes and marine resources.

Recent sports development replaced natural turf with artificial turf (also referred to as synthetic turf) due to its low maintenance requirements, cost savings, and greater access in all weather conditions^28^. For instance, in Japan, artificial turf fields increased by approximately 250 sports fields annually from 2014 to 2019 and approximately 3500 fields during 2000–2019^28^. Today, over 10,000 fields exist in the United States and over 13,000 in Europe^29^. These artificial turf fields contain rubber tips (RT) (also called crumb rubber, rubber granulates, and rubber granules) as an infill to keep the upright structure of artificial turf fibers and shock absorption of the field players. The materials for the RT are known for some health risks for the field players because some toxic chemicals and heavy metals contained in the RT could be ingested, inhaled, or dermally absorbed by the field players^29–37^. Apart from humans, leachate from RT layers was toxic for chicken embryos and water flea *Daphnia magna*^38^. Thus, although RT is not so-called “plastic,” we should consider RT as a marine MP in the broad sense because not only the safety of the players on the turf but also the safety of aquatic animals should be considered^39,40^.

In the context of sports and fisheries, two questions should be considered: whether artificial turf fields can be one of the sources of MP in other environments and whether RT impacts the health of aquatic animals, including marine resources. Sieber et al.^41^ reported that 357 t of RT was released from artificial turf into the environment in Switzerland in 2018. Kole et al.^42^ also estimated that the released rate of RT is 380–640 t/year in Denmark, 2300–3900 t/year in Sweden, and 2700–4500 t/year in The Netherlands. Although some studies have examined the actual migration of the RT from the sports field^34–36,39,40^, no studies have examined a relationship between the RT and fish activities. Therefore, it is crucial to clarify the influence of sports activities, including sports facilities, on marine ecosystems, marine resources, and seafood safety^43^.

This study aims to examine whether artificial turf sports fields could be a source of marine MP. First, we surveyed to examine whether RTs migrate from the field to the ditches surrounding the artificial turf sports field of International Christian University (ICU). Then, we collected sediments in the ditches and measured the amount of RT. Next, we examined whether fish ingest RT when RT is placed in the water. We used the goldfish *Carassius auratus,* a freshwater omnivorous fish with different body sizes, as an experimental model since goldfish is widely used as an experimental model fish^44^. We also used the wild Japanese crucian carp *Carassius auratus* subsp. 2. The RTs were given to fish with or without fish feed to examine whether the ingestion was active or incidental (accidental). Retention and elimination of RT in the intestine were also studied in goldfish.

## Results

### Migration of RT from the field to the ditch

**Figure 1:**
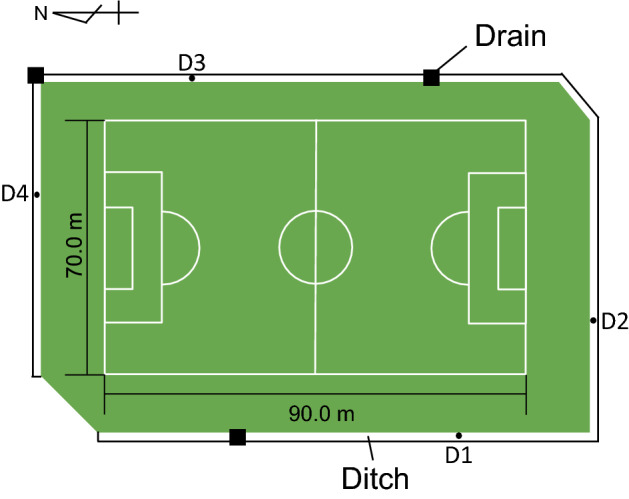
A schematic illustration of a ground plan of the artificial turf sports field of International Christian University. There are ditches along four sides of the field. Three small solid squares on the ditch indicate the drain that is connected to sewer pipes. D1-D4 indicate the locations where sediment samples were collected.

**Figure 2:**
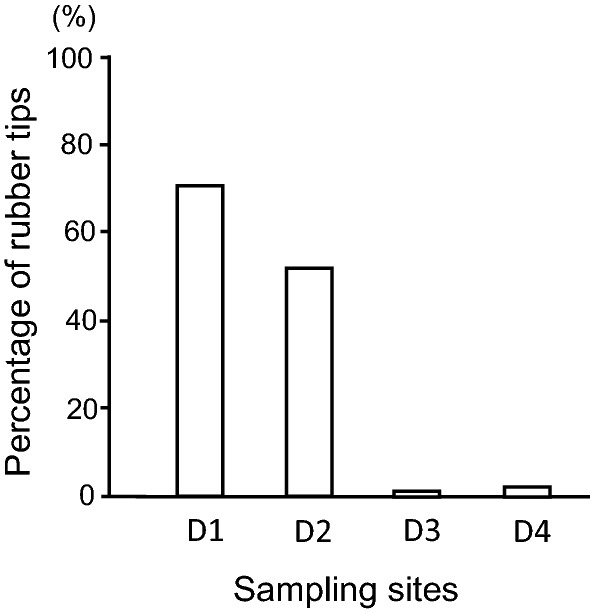
Fraction (%) of rubber tips in the sediment. D1–D4 indicate the four sites in the ditch where sediments were collected.

Sediments collected in the ditch surrounding the artificial turf field contained RT at all four sampling sites (Fig. 1). The sediments consisted of soil, stones, dead leaves, fibres of artificial turf, and RT. The fraction of RT (w/w) in the sediments varied among the sites: D1, 70.7%; D2, 50.7%; D3, 1.5%; D4, 2.1% (Fig. 2).

### Co-ingestion of RT and fish feed by goldfish

**Figure 3:**
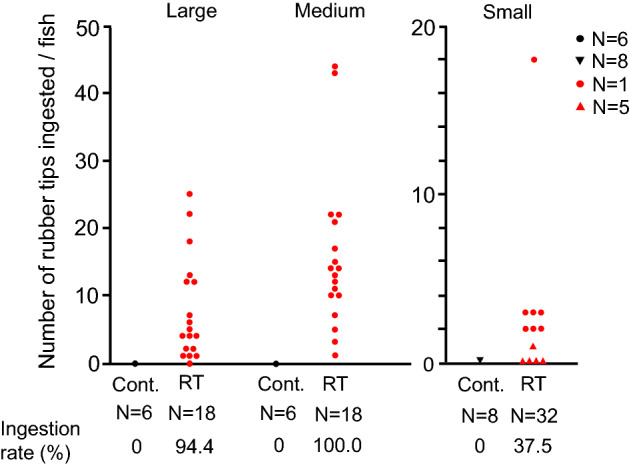
Co-ingestion of rubber tips (ICU) and fish feed by goldfish of different body sizes. Fish were fed a mixture of fish feed and rubber tips. The number of ingested rubber tips in the intestine was counted at dissection. Cont., fish of the control group fed with only fish feed; RT, fish fed with a mixture of fish feed and rubber tips.

Goldfish of three different body sizes (large, medium, and small) were given RT collected from the field of ICU and one of three sizes of fish feed depending on the body size of the fish. Ninety minutes after feeding, the fish were dissected. Ingested RT existed in the intestine in all body-size groups (Fig. 3). The ingestion rates of RT in large-, medium- and small-sized fish were 94.4, 100, and 37.5%, respectively. The number of RTs ingested varied from 0 to 25 (median, 5.5) for large size, from 1 to 44 (median, 13.5) for medium size, and from 0 to 18 (median, 0) for small size. No RT was observed in the intestine of the fish in the control groups fed only fish feed. The numbers of ingested RT were significantly higher in the experimental groups than in the respective control groups: large fish, *P* = 0.000561; middle fish, *P* = 0.000283; small fish, *P* = 0.0448 by the Mann–Whitney *U* test.

**Figure 4:**
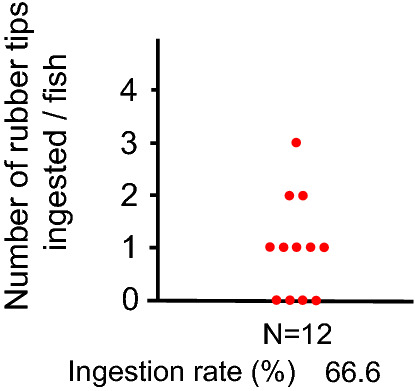
Co-ingestion of rubber tips (TGU) and fish feed by goldfish. Fish were fed a mixture of fish feed and rubber tips. The number of ingested rubber tips in the intestine was counted at dissection.

RTs collected from Tokyo Gakugei University (TGU) were also ingested by large body size goldfish, but the numbers were much smaller than those of the ICU (Fig. 4). The number of RTs ingested varied from 0 to 3 (median, 1.0).

**Figure 5:**
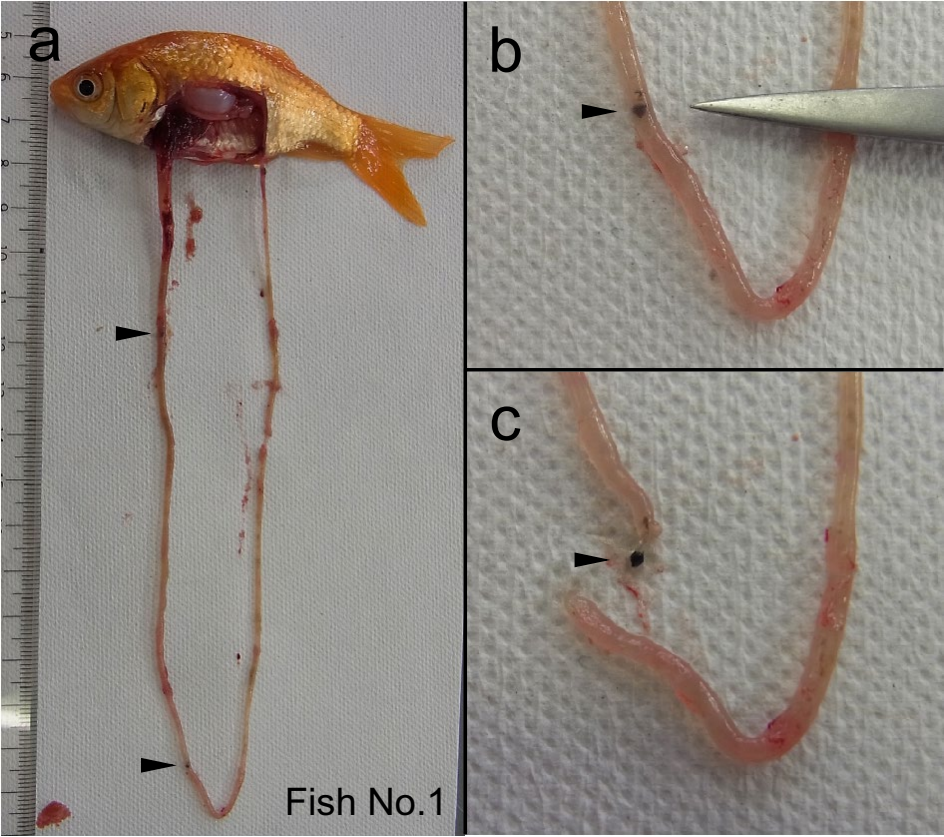
Rubber tips found in the intestine of goldfish (Fish No. 1). Fish were fed a mixture of fish feed and rubber tips. Two pieces of rubber tips were observed in the intestine of the fish at dissection. Arrowheads indicate rubber tips. Photographs (**b**) and (**c**) are closeup of the lower part of photograph (**a**).

**Figure 6:**
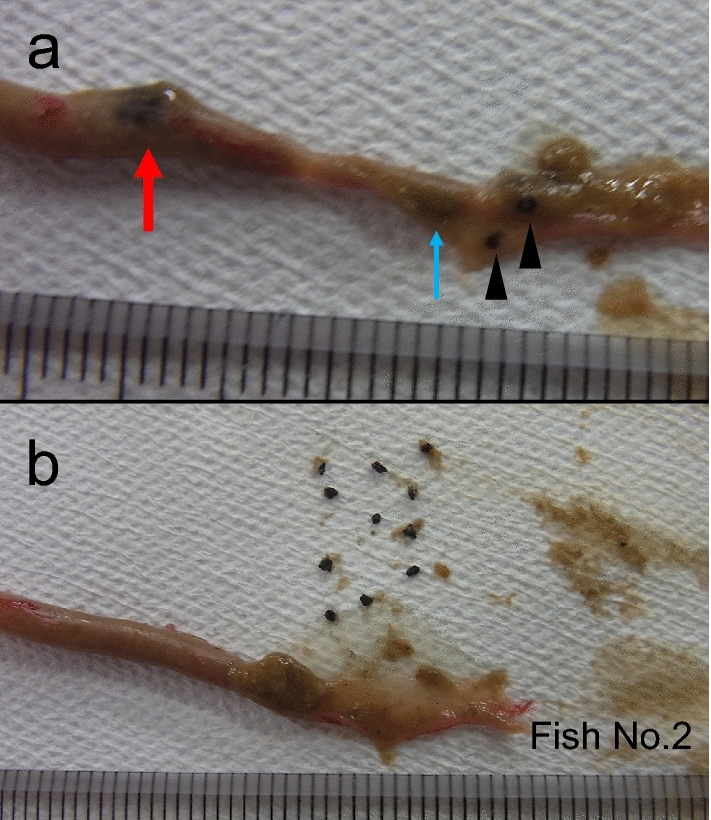
Rubber tips found in the intestine of goldfish (Fish No. 2). Sixteen pieces of rubber tips were found in the intestine in total. (**a**) A red arrow indicates a mass of eleven pieces of rubber tips. A blue arrow indicates a mass of three pieces of rubber tips. Arrowheads indicate one piece of rubber tip. (**b**) Eleven pieces of rubber tips came out from the intestine at the site indicated with a red arrow in photograph (**a**).

An additional experiment using small-medium body-size fish resulted in eight out of nine fish ingesting RT (ICU). The number of ingested RTs varied from 0 to 16 (median 1.0). For example, fish No. 1 ingested two RTs (Fig. 5), but fish No. 2 ingested 16 RTs (Fig. 6). Then, the number of ingested RTs in other fish was 2, 1, 1, 1, 1, 1, and 0.

### Active ingestion of RT by goldfish

**Figure 7:**
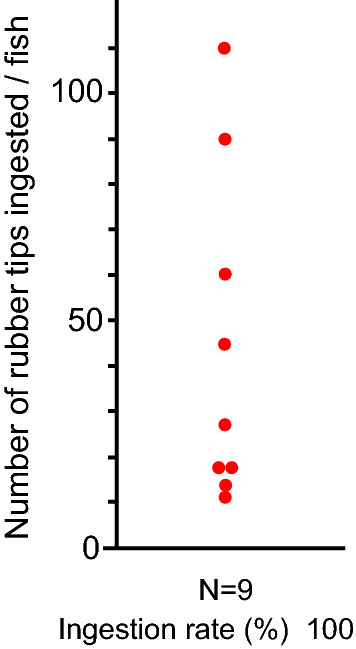
Active ingestion of rubber tips by goldfish. Goldfish ingested rubber tips in the absence of fish feed. The number of ingested rubber tips in the intestine was counted at dissection.

Large body size goldfish ingested RT actively in the absence of fish feed. By dissection, ingestion of RT was confirmed in all the tested goldfish (Fig. 7). The number of ingested RTs varied by individual from 11 to 110 (median, 27). This result indicates that goldfish ingested RT actively, not unintentionally, with feed.

### Retention and elimination of ingested RT in the intestine of goldfish

**Figure 8:**
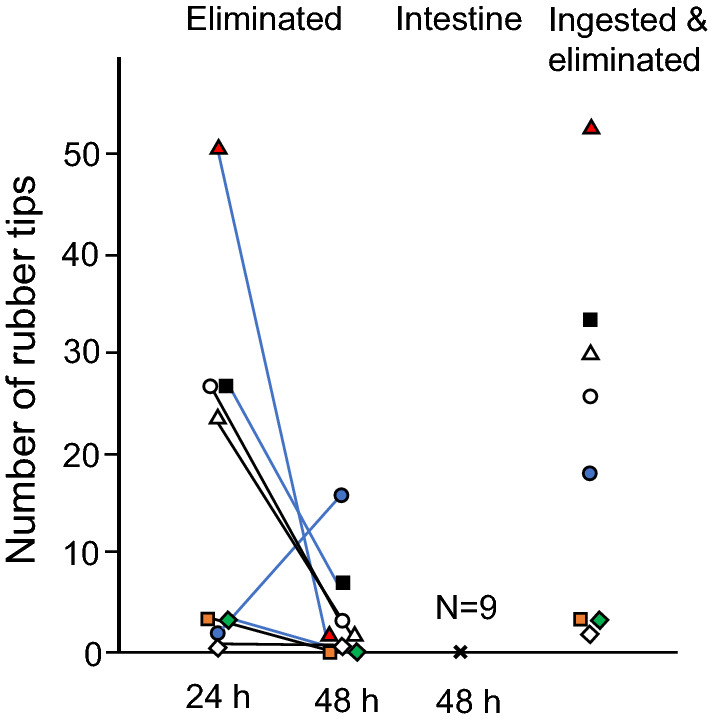
Retention and elimination of ingested rubber tips in the intestine of goldfish. Eight out of nine fish ingested and eliminated rubber tips. Each symbol indicates the number of rubber tips collected in the aquaria. The same symbols indicate the same individuals. There were no rubber tips in the intestine of any of the fish at 48 h (indicated with x).

Large body size fish were given RT and kept for 90 min and then transferred individually to different aquaria. Eliminated RT was found in the feces and on the bottom of the aquaria of eight out of nine aquaria at 24 or 48 h after RT feeding (Fig. 8). Seven fish eliminated RT within 24 h, and five fish eliminated RT within 48 h. The number of ingested and eliminated RT in eight fish varied 2–53 (median, 22). The number of eliminated RT varied from 1 to 51 (median 13.5) at 24 h and 0–16 (median, 2.0) at 48 h. When fish were dissected at 48 h (Day 6), no RT was observed in the intestine of the nine fish.

### Ingestion of RT by wild crucian carp

Wild juvenile crucian carp ingested RT (ICU) when the fish were given RT with or without fish feed. RT were collected from the intestine of fish given a mixture of RT and fish feed. The number of RTs ingested by fish was 2, 2, 1, 0, 0 (N = 5). RT were also collected from fish given only RT. The number of RTs ingested by fish was 1, 1, 0, 0, 0 (N = 5). No RT was found in control fish which were given only fish feed (N = 6). The number of ingested RT was significantly higher in a group given with a mixture of RT and feed than in the control group (*P* = 0.0359 by the Mann–Whitney *U* test). The number of ingested RT between control fish and fish given only RT was not significantly different (*P* = 0.102 by the Mann–Whitney *U* test).

## Discussion

The results of the present study indicate that the RT of artificial turf sports fields migrated to the ditches connected to sewer pipes, and therefore, the artificial turf sports field could be one of the sources for marine MP. The present study also demonstrated that the RT in the water was ingested by fish, goldfish and wild crucian carp. This is the first demonstration of the active ingestion of RT of artificial turf fields by fish, and the results showed the possibility that RT released to aquatic environments could be ingested by wild fishes.

The natural factors, such as the wind and the outflow of the rainwater or the malformed surface caused by sports activity for a long period of time, greatly contribute to the microscale site variance for the migration of RT from the artificial turf field to the surrounding ditch. The sediments from the two sampling sites consisted of more than ten times the RT (w/w) compared with the other two sites.

Nonetheless, since the ditch is connected to a sewer pipe, the RTs in the ditch are most likely sent to a river near the university or to a sewage treatment plant. Fibers of artificial turf were also found in the sediment in this survey, although quantification was not made in the present study. There are two types of sewage systems in Japan. An old type of sewage system transports both wastewater and rainwater to the sewage treatment plant. A new system transports wastewater and rainwater separately; wastewater is sent to the sewage treatment plant, and rainwater is sent directly to rivers. The pathways of MP from sewage treatment plants to the environment are different by the size of MP; small and light MP are emitted with treated water runoff from the plant into the river, and heavier MP precipitate into the sewage sludge, which is used for an agricultural soil amendment and fertilizer in landfills^45,46^. In fact, MPs were found in agricultural soil to which sewage sludge has been applied^11^. The MP originating from sewage sludge could be washed out from the agricultural soil by wind and rain, resulting in the spreading of MP into the aquatic environment. In the case of RT of the artificial turf field, it could be sent to rivers with rainwater or spread to the soil with sewage sludge via sewage treatment plants. Fibers of artificial turf released from the sports field would also go to rivers or the sewage treatment plant. In contrast, the artificial turf fiber could enter the river system with treated water runoff because it floats in the water.

The release of the RT from the artificial turf field was recognized in some European countries^41,42^. As the installation of artificial turf fields in Japan has increased^28^, these fields could become significant sources of MP in the aquatic environment. Considering the European examples, it is vital to take some mitigation action for the RT from the sports fields. One possible way to mitigate the spread of RT is to install a device to prevent the migration of RT from ditch to sewer pipes. Another option is to use natural products, such as cork, coir (coconut-derived material), or olive seed, as infill for sports fields instead of RT^32,39,40^. Infills of plant origin are biodegradable and thus would not result as MP even if they are released into the environment. However, natural tips are more expensive than RT, and the durability of natural tips in the field is still unknown (personal communication from a Japanese company A.

Wild fish species larger than goldfish and crucian carp could ingest more RT in the natural environment because the goldfish in this study actively ingested RT from artificial turf fields, even though there was considerable individual variation in the number of ingested RT. The reason for the considerable variation is unknown. High ingestion rates of RT were observed in larger goldfish, probably because larger fish have strong suction power. Active ingestion of RT in goldfish would occur regardless of the manufacturer because of the similar result between two different companies, a Japanese company B and a Japanese company C in goldfish. However, the goldfish may have some preference because there was a five-fold difference in the ingestion rates between the RTs from the two companies. The reason why goldfish discriminated the RT of TGU from that of ICU is unknown.

Interestingly, goldfish actively ingested RT without feed as well as RT with feed. These results indicate that ingestion was not accidental or unintentional. Xiong et al.^24^ demonstrated that goldfish ingested polyethylene fragments only in the presence of fish feed. The same study also reported that ingestion rates were different among the shape and color of polyethylene fragments. Since RT for artificial turf fields has a round shape, dark color, and unique softness, goldfish could easily recognize RT as a food. When mummichog *Fundulus heteroclitus* and fathead minnow *Pimephales promelas* were exposed to a high dose of tire crumb rubber (6.0 g/L) for seven days*,* the tire crumb rubber was ingested by fish, accumulated in the intestine and showed toxicity and increased liver cytochrome P450-1A enzyme activity and glutathione S-transferase activity^21^. Although we did not examine damage to goldfish by RT, some fish ingested a large amount of RT, which may cause intestinal obstruction. Similar to the study by LaPlaca and van den Hurk^21^, the present study also indicated a high possibility that wild fish ingest RT when released into the aquatic environment.

The short retention period of ingested RT does not seem to cause serious damage to fish. The ingested RT in this study was eliminated within two days in all the goldfish examined, although we did not examine the re-ingestion of eliminated RT. This retention period of RT is comparable to that of polyethylene fragments (72 h)^24^. The round shape and softness of RT could be easier to move in the digestive tract compared to the rough and rigid polyethylene fragments. However, it may be long enough for the RT to go into the food web when fish are preyed on by predators.

In conclusion, the present study demonstrated that a sports facility has the potential to be a source of marine MP, and the RT of artificial turf fields could be ingested by fish when it is released into the aquatic environment. Sports is not only an important activity from educational, social, and commercial points of view, but its development is also essential for human well-being, similar to other artistic activities. Furthermore, sharing common awareness among society on the influence of sports activity on the environment is important for sustainable fisheries^43^.

## Materials and methods

### Statements

We report our study in accordance with ARRIVE guidelines.

### Structure of artificial turf of ICU

A schematic illustration of a ground plan of the artificial turf sports field of the ICU is shown in Fig. 1. This artificial turf was installed in 2013 by Japanese company B. The field is surrounded by ditches, and there are three drains that connect to sewer pipes. The artificial turf field of TGU was installed in 2011 by Japanese company C.

### Characterization of rubber tips of artificial turf field of ICU and TGU

RT were collected from the artificial fields of ICU and TGU. RT for the artificial turf field of ICU were made of residual part of rubber for making tires, window frames and windshields of automobiles. RT of ICU consists of a mixture of EPDM (ethylene-propylene-diene) and SBR (styrene-butadiene rubber) (personal communication from a Japanese company B). The RT of TGU was made of rubber of the residual part of rubber for making tires, window frames, etc. (personal communication from a Japanese company C). Information on raw material of the RT was not manifested.

RT collected from the fields (ICU and TGU) was sieved to estimate the particle sizes. The RT of the ICU varied from 0.053 to 3.35 mm, and that of TGU varied from 0.212 to 3.35 mm. The specific gravity of the RT was obtained as follows: A certain amount of RT was weighed and poured into a 10 ml graduated cylinder containing some water. The total volume of the RT was obtained by measuring the rise in the meniscus of the water. The specific gravities of the tested RT were 1.28 (ICU) and 1.28 (TGU). Elemental analyses of RT (ICU and TGU) were conducted using micro-PIXE line analysis^47^, and calcium, sulfur, zinc, and iron were detected, but lead was under the detection limit from the RT of both ICU and TGU.

### Sampling of sediments in the ditches of the field

To examine the migration of RT from the field to the ditches, approximately 200 g of sediments in the ditches was sampled at four different sites, D1–D4 (Fig. 1), in the ICU. The ditch surrounding the field is made by connecting U-shaped concrete blocks and concrete lids. The inner width, length, and depth of the block are 24, 60, and 24 cm, respectively. The size of the lid is 33 × 60 × 4.5 cm with 1.5 × 9.0 cm snicks at short sides, which make an opening of the ditch of 3.0 × 9.0 cm size between two lids.

Each sample was weighed (wet weight) and washed with water using a fine sieve to remove the soil. After the removal of the soil, the sediment was dried, and RT was collected manually. The collected RT was weighed, and the percentages of RT in the sediments were calculated (weight/weight).

### Goldfish and crucian carp

A common variety of goldfish *C. auratus* of different sizes were obtained from a fish merchant in Saitama Prefecture and from a pet shop in Tokyo and then kept in the ICU. Approximately 200 fish of four different sizes (large, body weight (BW) ~ 100 g; medium, BW, ~ 30 g; small-medium, BW, ~ 15 g; small, BW ~ 4.0 g) were kept in three 800-L stock tanks maintained at 20 °C under a 16-h light/8-h dark (16 L/8 D) photoperiod (lights on at 06:00). Small body size fish were kept in a floating cage in one of the stock tanks. The fish were fed commercial floating goldfish feed (Itosui) once a day ad libitum. The fish stock tanks had circulation filtration systems equipped with sand filters. The filter was cleaned every week to maintain the water quality. The health condition of the fish was judged by their appetite. All the experimental fish (mixed sex) in the present study were kept in stock tanks for over two weeks before they were used for experiments. A total of 127 goldfish were used for the present study. The sample size of each experiment was determined by the results of preliminary experiments. Our preliminary survey confirmed that the fish feed we used did not contain RT-like substances. Therefore, the sample sizes of the control groups (goldfish) were smaller than those of the experimental groups to sacrifice fewer fish. All goldfish and crucian carp experiments were conducted in the ICU.

Approximately 30 wild juvenile crucian carp *C. auratus* subsp. 2 weighing 1.4–4.6 g were obtained from a fish merchant in Saitama Prefecture and kept in an 800-L stock tank in the same conditions as that for goldfish. A total of 16 crucian carp were used for the present study.

For the experiments, fish were transferred from the stock tanks to experimental 60-L glass aquaria, which were maintained at 20 °C under a 16-h light/8-h dark (16 L/8 D) photoperiod (lights on at 06:00). The experimental aquaria had a running water system, and dechlorinated tap water was added at 20 ml/min. Plastic box filters were also set to each experimental aquarium to maintain water quality. When stock fish were transferred to experimental aquaria, fish were randomly allocated to the aquaria. All the methods for using goldfish and crucian carp were performed in accordance with the guidelines of the Animal Experimentation Committee of International Christian University. The conduct of the present study was approved by the Animal Experimentation Committee of International Christian University.

### Co-ingestion of feed and RT by goldfish of three different body sizes

We examined whether RT are ingested by goldfish with feed and whether the body size of fish affects the ingestion of RT using three different body sizes of fish, large, medium, and small. First, we conducted an experiment using large body size fish (N = 24; BW, 91.9 ± 21.6 g, mean and SD). Three goldfish of large body size were transferred from the stock tank to the experimental 60-L glass aquarium and kept for three days for acclimation of the environment and sinking fish feed. Fish were fed 3.0 g of large-size feed (Japan Pet Design Co. Ltd.) once a day. On the fourth day, fish were fed a mixture of RT collected from the field (ICU, 300 mg) and large feed (3.0 g). Control fish were fed only fish feed. At 90 min after feeding, the fish were transferred to a pail containing 0.05% 2-phenoxyethanol solution and deeply anesthetized. After body weight measurement, fish were dissected. We observed the intestine to determine whether RT was ingested. When RT was observed in the intestine, we collected the tips and counted the number of tips in each fish. The experimental tests were repeated eight times, and the data were combined.

Second, we conducted an experiment using medium body size fish (N = 24; BW, 30.4 ± 12.4 g). Three goldfish of medium body size were transferred from the stock tank to the experimental 60-L glass aquarium and kept for three days for acclimation. Fish were fed 1.0 g of medium-size feed (Kyorin) once a day. On the fourth day, fish were fed a mixture of RT (ICU, 300 mg) and medium feed (1.0 g). Control fish were fed only fish feed. At 90 min after feeding, fish were anesthetized and dissected, and then the intestine was observed as described above. The experimental tests were repeated eight times, and the data were combined.

Third, we conducted an experiment using small body size fish (N = 40; BW, 4.4 ± 1.5 g). Four goldfish of small body size were transferred from the stock tank to the experimental 60-L glass aquarium and kept for three days for acclimation. Fish were fed 0.5 g of small-size feed (Kyorin) once a day. On the fourth day, fish were fed a mixture of RT (ICU, 300 mg) and small feed (0.5 g). Because of the small size of fish, RT of small size particles (212–500 µm) were collected with sieves and used for the tests. Control fish were fed only fish feed. At 90 min after feeding, fish were anesthetized and dissected, and then the intestine was observed as described above. The experimental tests were repeated ten times, and the data were combined.

In the first three experiments, all three control groups showed no ingestion of RT. From the results of the three experiments, it was clear that our experimental system was not contaminated with RT. Therefore, we omitted making control groups for further experiments to decrease the number of fish sacrificed from the standpoint of fish welfare.

Fourth, we examined whether RT collected from TGU was ingested by goldfish. We conducted an experiment using large body size fish (N = 12; BW, 140.3 ± 27.0 g). Three goldfish of large body size were transferred from the stock tank to the experimental 60-L glass aquarium and kept for three days for acclimation. Fish were fed 3.0 g of large-size feed once a day. On the fourth day, fish were fed a mixture of RT (TGU, 300 mg) and large feed (3.0 g). At 90 min after feeding, fish were anesthetized and dissected, and then the intestine was observed as described above. The experimental tests were repeated four times, and the data were combined.

We conducted an additional experiment with a similar design to those of the four experiments to take photographs of the fish and RT using fish of small-medium body size (N = 9; BW, 12.8 ± 2.7 g). Three fish were transferred from the stock tank to the experimental 60-L glass aquarium and kept for two days for acclimation. Fish were given 0.5 g of medium-size feed once a day. On the third day, fish were given a mixture of RT of ICU (30 pieces; size 0.5–1.0 mm) and medium feed (0.5 g). At 60 min after feeding, fish were anesthetized and dissected, and photographs of RT in the intestine were taken. The experimental tests were repeated three times, and the data were combined.

### Active ingestion of RT by goldfish

We examined whether goldfish actively ingest RT when RT are given without fish feed using large body size fish (N = 9; BW, 122.4 ± 20.8 g). Three fish were transferred from the stock tank to the experimental 60-L glass aquarium and kept for three days for acclimation. Fish were fed 3.0 g of large-size feed once a day. On the fourth day, fish were given 300 mg of RT (ICU) on the bottom of the aquarium. At 90 min after the placement of RT, fish were anesthetized and dissected, and then the intestine was observed as described above. The experimental tests were repeated three times, and the data were combined.

### Retention and elimination of ingested RT in the intestine of goldfish

We examined how long RT was retained in the intestine using large body size goldfish (N = 9; BW, 101.6 ± 11.4 g). Three goldfish were transferred from the stock tank to the experimental 60-L glass aquarium and kept for three days for acclimation. Fish were fed 3.0 g of large-size feed once a day. On Day 4, fish were given 1.0 g of RT (ICU). At 90 min after the placement of RT, each fish was individually transferred to three experimental 60-L glass aquaria. Then, each fish was fed 1.0 g of the feed. At 24 and 48 h (Day 5 and Day 6) after the transfer, we collected feces from fish and some water from the bottom of the aquaria. We observed whether RT was eliminated from the fish into the aquaria. When RT was observed in the feces and the bottom of the aquarium, we collected the RT and counted the number of RT. On Day 5, after the RT observation, each fish was fed 1.0 g of the feed. On Day 6, after RT observation in feces and water, the fish were anesthetized and dissected. We observed whether the intestine retained RT. The experimental tests were repeated three times, and the data were combined.

### Ingestion of RT by wild crucian carp

We examined whether wild Japanese crucian carp ingest RT. The experiment was conducted using juvenile crucian carp (N = 16, BW, 2.8 ± 0.9 g). Sixteen fish were transferred from the stock tank to three experimental 60-L glass aquaria (5 or 6 fish per aquarium) and kept for six days for acclimation. Fish were fed with 0.2 g of small-size feed once a day. On the seventh day, fish were fed a mixture of RT (ICU, 30 mg) and the small feed (0.2 g) or RT alone (30 mg). Because of the small size of fish, RT of small size particles (212–500 µm) were collected with sieves and used for the test. Control fish were fed only fish feed (0.2 g). At 6 h after feeding, fish were anesthetized and dissected, and then the intestine was observed as described above.

## Data Availability

The dataset supporting the conclusions of this article is included with the article.

## Abbreviations

RT: Rubber tip
MP: Microplastic
ICU: International Christian University
TGU: Tokyo Gakugei University
